# Reducing off-target effects of DdCBEs by reversing amino acid charge near DNA interaction sites

**DOI:** 10.1038/s41422-024-01028-w

**Published:** 2024-09-10

**Authors:** Long Xie, Yaqi Cao, Di Li, Mengxue Ma, Danrong Jiao, Hu Feng, Zhenrui Zuo, Erwei Zuo

**Affiliations:** Shenzhen Branch, Guangdong Laboratory for Lingnan Modern Agriculture, Key Laboratory of Synthetic Biology, Ministry of Agriculture and Rural Affairs, Agricultural Genomics Institute at Shenzhen, Chinese Academy of Agricultural Sciences, Shenzhen, Guangdong China

**Keywords:** Molecular biology, Biological techniques

Dear Editor,

DddA_tox_-derived cytosine base editors (DdCBEs) can mediate precise, CRISPR-independent editing of mitochondrial DNA (mtDNA), but were recently shown to potentially introduce extensive and non-trivial off-target effects throughout the genome,^1,2^ presenting a significant obstacle for research applications and an obvious safety issue for use in clinical therapies of hereditary mitochondrial disorders. Here, utilizing the crystal structure of DddA_tox_ in complex with DNA, we predicted DNA-binding sites on the DddA_tox_ protein surface and generated variants by converting positively charged residues at these sites to negatively charged amino acids. By modifying the DNA-binding capability of DddA_tox_ through charge reversal, especially through K1402D or K1402E conversions, we could generate split-architecture DdCBEs with high on-target editing efficiency at multiple sites in mtDNA and significantly fewer off-target effects in both mtDNA and whole genome of mice, ~400 times lower than wild-type (WT) DdCBE (DdCBE^WT^). This study establishes K1402D/K1402E-DdCBE (DdCBE^K1402D/E^) editors as efficient, high-fidelity, and relatively safe research tools for mitochondrial diseases that warrant exploration for therapeutic applications, and demonstrates a strategy for reducing off-target effects in DddA_tox_ family base editors.

Deaminase and DNA-binding activities are both essential for DNA modification by cytosine base editors. To reduce off-target effects of DddA_tox_ while retaining its catalytic efficiency, we hypothesized that altering positively charged amino acids on the protein surface (i.e., arginine (R), histidine (H), and lysine (K) residues) that interact with negatively charged DNA could reduce the DNA-binding affinity of DddA_tox_ (Supplementary information, Fig. S1), potentially decreasing off-target editing activity. Based on the solved three-dimensional (3D) structure of DddA_tox_^3^ (Fig. 1a), we identified five such candidate sites (H1345, K1402, R1403, K1420, K1424; Fig. 1b), among which, K1402, R1403, K1420, and K1424 were located in the C-terminal region of the previously established G1333 or G1397 cleavage sites. Preliminary AlphaFold2 predictions of variants carrying negatively charged aspartic acid (D) or glutamic acid (E) at these five sites suggested that H1345D/E, K1402D/E, R1403D/E, K1420D/E, or K1424D/E conversions were unlikely to significantly alter the protein structure beyond the charge at those sites (Supplementary information, Fig. S2b, c).

**Fig. 1:**
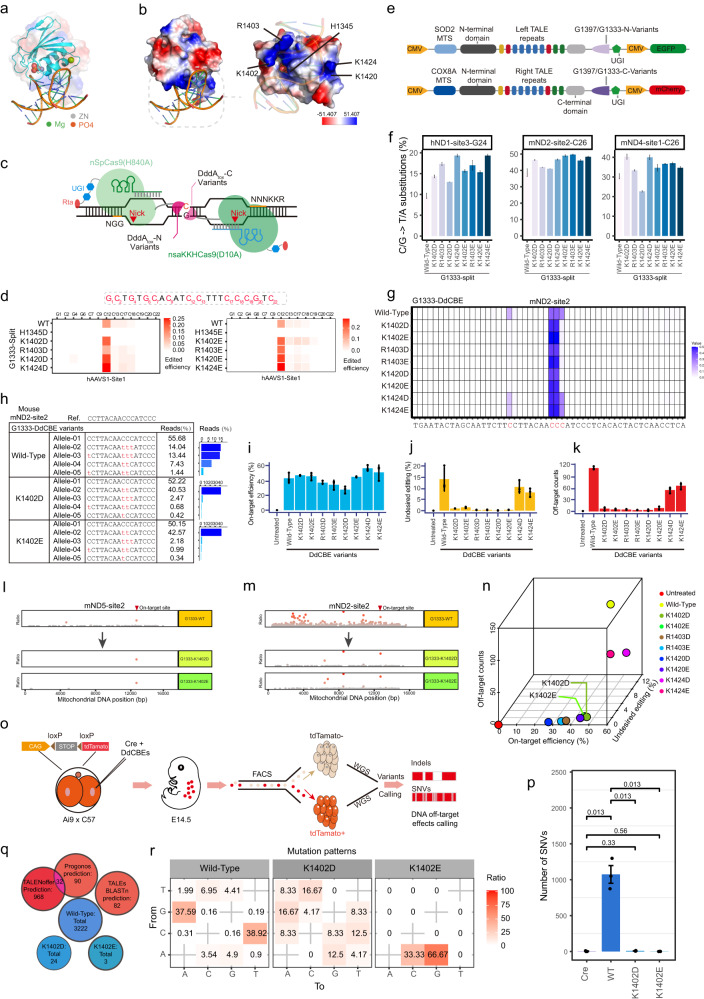
Minimizing off-target effects of DdCBEs by reversing amino acid charges in proximity to DNA interaction sites. **a** Secondary crystal structure of the DddA_tox_ deaminase in complex with DNA. **b** Space filling model of DddA_tox_ protein with negatively charged surface residues shown in red and positively charged residues in blue. **c** Architectures of split-DddA_tox_-Cas9 fusions. DddA_tox_-N and DddA_tox_-C termini were fused to nSpCas9 (H840A) and nSaKKHCas9 (D10A), respectively. NGG, protospacer adjacent motif (PAM) for nSpCas9 (H840A); NNNKKR, PAM for nsaKKHCas9 (D10A). **d** Heat maps of C/G-to-T/A conversion frequencies for G1333-split-DddA_tox_-Cas9 D/E variants split at the hAAVS1 sites. **e** Plasmid structures of DdCBE variants for mitochodrial base editing. **f** Editing efficiencies of G1333-DdCBE variants in human and mouse mitochondrial genome sites. **g**, **h** Detailed characteristics (**g**) and editing alleles (**h**) of G1333-DdCBE variants at mND2-site2. **i** Frequencies of C/G-to-T/A conversion within the target sequence at mND5-site2. **j** The undesired C/G-to-T/A conversion frequencies of different G1333-DdCBE variants at mND5-site2. Undesired editing: other edited positions within the editing windows, aside from the targeted site “C”. **k** The off-target editing counts of various G1333-DdCBE variants at mND5-site2. **l**, **m** The off-target distributions of G1333-DdCBE variants at mND5-site2 (**l**) and mND2-site2 (**m**). **n** 3D scatter plot comparing editing efficiencies, off-target editing counts, and undesired C/G-to-T/A in-window conversions of different DdCBE variants. **o** Schematic diagram of genome-wide off-target analysis by two-cell embryo injection (GOTI). **p** Quantification of single nucleotide variants (SNVs) resulting from off-target editing for WT and split DdCBEs variants. The data of Cre samples were derived from our previous study.^7^ **q** Off-target locus prediction by three different algorithms showed no overlap with off-target sites detected in each DdCBE group and there was no overlap among WT and DdCBE variants in all replicates (*n* ≥ 3). **r** SNV conversion patterns for all replicates (*n* ≥ 3) of DdCBE^WT^ and DdCBE^K1402D/E^ variants. All *P* values were calculated by two-sided Student’s *t*-tests. *n* ≥ 3 replicates were used in all experiments.

We employed the well-established Split-DddA_tox_-Cas9 fusion architecture^4,5^ to initially assess the catalytic activity of these variants. In split architectures, the DddA_tox_-N half is fused to the N-terminus of nickase Cas9 (nCas9), while the DddA_tox_-C half is fused to the N-terminal region of an orthogonal nickase *Staphylococcus aureus* Cas9 variant (nSaKKH-Cas9) (Fig. 1c; Supplementary information, Fig. S3a). Various sgRNA pairs can be used to examine the cytosine base editing characteristics of each DddA_tox_ variant at different target sites. We therefore introduced D or E mutations at each of the abovementioned sites (H1345D/E, K1402D/E, R1403D/E, K1420D/E, or K1424D/E) in DddA_tox_ variants split at either G1333 or G1397, to investigate possible differences in preferential editing between these forms, resulting in 20 split variants. The split variant halves were then fused to the Cas9 variants following the strategy described above, and two plasmids carrying the two Split-DddA_tox_-SaKKH-Cas9/Cas9 halves were co-transfected into HEK293T cells along with plasmids harboring their corresponding sgRNAs and mCherry or eGFP reporters for nSaKKH-Cas9 or nSpCas9, respectively. Cells were sorted by dual fluorescence at 48 h post transfection for DNA extraction, target site amplification, and Illumina sequencing.

Sequencing analysis of the target sites revealed that the K1402D/E, R1403D/E, K1420D/E, and K1424D/E variants exhibited cytosine base editing characteristics similar to WT split-DddA_tox_-Cas9. However, the H1345D/E variant showed lower C/G-to-T/A conversion at most target sequences (Fig. 1d; Supplementary information, Fig. S3b–d). We also noted that several variants preferentially edited cytosines between the 10th and 15th bases in the target sequence, with the highest frequency at position 11 and significantly lower cytosine editing at position 14. Furthermore, the K1402D/E and R1403D/E variants displayed C/G-to-T/A conversion frequencies comparable to WT split-DddA_tox_-Cas9 in both the G1333 and G1397 forms, whereas the K1424D/E variants had slightly higher conversion frequency. Further analysis indicated that the K1402D/E, R1403D/E, K1420D/E, K1424D/E, and WT DdCBEs preferentially targeted cytosines in the “TC” sequence context, with K1402D/E, R1403E, and K1420D/E displaying higher stringency than other variants at sites within the target window (Supplementary information, Fig. S3e). These results indicated that the K1402D/E, R1403D/E, K1420D/E, and K1424D/E variants retained similar base editing properties to WT split-DddA_tox_-Cas9 and warranted further assessment.

As these variants retained the WT Split-DddA_tox_-Cas9 editing characteristics at target sites in the genome, we next evaluated their cytosine editing in the mitochondrial genome by fusing the K1402D/E, R1403D/E, K1420D/E, or K1424D/E split variants with the C-terminus of TALE modules (Fig. 1e). Briefly, each pair of DdCBE variants targeting different mitochondrial loci were co-transfected into HEK293T or mouse N2a cells, and these cells were incubated for 48 h to allow EGFP/mCherry co-labeling and then isolated by fluorescence-activated cell sorting (FACS). Mitochondrial target sites in each cell line were then amplified by PCR for Illumina sequencing and analysis of editing characteristics (Supplementary information, Fig. S4a).

We observed that editing efficiencies varied significantly among mitochondrial target sites for DdCBE variants in the G1397 form, with only DdCBE^K1424D/E^ maintaining consistent editing efficiency across most sites and edited C positions, comparable to DdCBE^WT^. In the hND1-site2-C29 context, G1397-DdCBE^WT^ displayed an editing efficiency of 14% ± 1.02%, while the G1397-DdCBE^K1402D/E^ variants exhibited significantly higher editing efficiency, achieving 25% ± 0.94% and 25% ± 1.3%, respectively (Supplementary information, Fig. S4b, d). By contrast, the G1397-DdCBE^K1420D/E^ variants showed markedly lower editing efficiencies, both < 2%. At mND4-site1, the DdCBE^WT^ had an editing efficiency of 25% ± 1.22% (Supplementary information, Fig. S4b). However, the performances of DdCBE variants varied widely, with DdCBE^K1420D^ showing no editing activity (0%) and DdCBE^K1424E^ reaching an editing efficiency of 8% ± 1.3% (Supplementary information, Fig. S4b). This variable editing efficiency was also observed across other G1397-DdCBE target sites, including hND5-site1, mND1-site2, mND4-site2, and mND6-site1.

In the G1333 split form, the DdCBE^K1402D/E^, DdCBE^R1403D/E^, and DdCBE^K1420D/E^ variants mostly showed editing efficiencies within 5% lower, or in some cases higher, than DdCBE^WT^. At mND2-site2-C26, G1333-DdCBE^WT^ displayed 33.7% ± 2.2% editing efficiency, while G1333-DdCBE^K1402D/E^, G1333-DdCBE^R1403D/E^, G1333-DdCBE^K1420D/E^, and G1333-DdCBE^K1424D/E^ achieved editing efficiencies of 45.9% ± 2.2%, 50.2% ± 0.2%, 42% ± 0.1%, 48.3% ± 0.2%, 40.2% ± 0.2%, 46.4% ± 0.2%, 45.2% ± 0.2%, and 47.6% ± 0.2%, respectively. This trend was also observed at other sites, including hND1-site1, hND1-site3, mND4-site1, and mND6-site2 (Fig. 1f; Supplementary information, Fig. S4c). These findings suggest that the G1333 form enables comparable editing efficiencies between the WT DddA_tox_ and its variants, making it more suitable for investigating the off-target effects of DdCBE variants.

Notably, we also observed that G1333-DdCBE^K1402D/E^, G1333-DdCBE^R1403D/E^, and G1333-DdCBE^K1420D/E^ had narrower editing windows than G1333-DdCBE^WT^. At mND4-site1, G1333-DdCBE^WT^ could edit the C20 base, whereas G1333-DdCBE^K1402D/E^, G1333-DdCBE^R1403D/E^, and G1333-DdCBE^K1420D/E^ variants catalyzed no detectable editing at this site (Supplementary information, Fig. S4e). Similarly, at mND2-site2, the G1333-DdCBE^K1402D/E^, G1333-DdCBE^R1403D/E^, and G1333-DdCBE^K1420D/E^ variants only mediated editing at C26 and C27, while DdCBE^WT^ showed editing effects at C19, C27, C28, and C29 (Fig. 1g). Analysis of edited alleles at mND2-site2 revealed edits primarily in allele-02, allele-03 and allele-04 in G1333-DdCBE^WT^ assays, accounting for 14.04%, 23.44%, and 7.43% of total reads, respectively. By contrast, edited reads were predominantly represented as a single edited allele in tests of G1333-DdCBE^K1402D/E^, G1333-DdCBE^R1403D/E^, and G1333-DdCBE^K1420D/E^ (Fig. 1h; Supplementary information, Fig. S4f), comprising 37.67–44.80% of the total reads. These results suggested that G1333-DdCBE fusions harboring the K1402D/E, R1403D/E, or K1420D/E variants had a narrower editing window than G1333-DdCBE^WT^, thus highlighting their increased fidelity.

To evaluate the off-target effects of these G1333-DdCBE variants in mtDNA, we used a pair of TALEs along with the K1402D/E, R1403D/E, K1420D/E, or K1424D/E DddA_tox_ variants to construct G1333-DdCBEs that could induce nonsense mutations in the mouse mitochondrial ND5 (mND5-site2) gene^1^ (Supplementary information, Fig. S5a). After transfecting plasmids harboring the 8 respective G1333-DdCBE variants into murine N2a cells and incubating cells for 48 h, we assessed C-to-T editing efficiency at the mND5-site2 (Supplementary information, Fig. S5b). Illumina sequencing analysis indicated that six variants retained similar editing efficiencies to G1333-DdCBE^WT^ at the on-target C_9_ ND5 location, including G1333-DdCBE^K1402D/E^, G1333-DdCBE^R1403D/E^, and G1333-DdCBE^K1420D/E^ (Fig. 1i; Supplementary information, Fig. S5d). We also noted that G1333-DdCBE^K1402D/E^, G1333-DdCBE^R1403D/E^, and G1333-DdCBE^K1420D/E^ variants had lower undesired editing (other edited positions within the editing windows, aside from the targeted site “C”) efficiencies than G1333-DdCBE^WT^ at site G_14_ (Fig. 1j; Supplementary information, Fig. S5c), which is potentially related to the narrower editing window that we identified above.

We next sequenced the full-length mtDNA from N2a cells expressing the respective DdCBE variants and quantified C/G-to-T/A conversion events. This analysis detected significantly fewer off-target effects in the mitochondrial genomes of N2a cells transfected with the G1333-DdCBE variants compared to G1333-DdCBE^WT^, which had 110 ± 3.4 total off-target edits (Fig. 1k). Notably, 5 variants (G1333-DdCBE^K1402D/E^, G1333-DdCBE^R1403D^, G1333-DdCBE^K1420D/E^) each had fewer than 10 off-target edits (Fig. 1k, l). Furthermore, the G1333-DdCBE^K1424D/E^ variants had slightly higher editing efficiency than G1333-DdCBE^WT^, although each catalyzed editing at ~50 off-target sites (Fig. 1i, k).

Closer examination of the off-target editing sites revealed that the G1333-DdCBE^WT^ and G1333-DdCBE^K1424D/E^ variants which catalyzed the most off-target base editing showed clear preferential targeting of “TC” motifs (Supplementary information, Fig. S5e), and primarily mediated C- > T/G- > A substitutions. In comparison, variants that introduced fewer off-target effects showed no consistent alternative targets or substitution types (Supplementary information, Fig. S5e, f), possibly due to the limited number of mutations available for analysis. Further analysis of the off-target distribution across the mitochondrial genome indicated that these events were randomly distributed (Fig. 1l, m; Supplementary information, Fig. S5g, h), and the off-target sites had no obvious TALE-related characteristics (Supplementary information, Fig. S5i), implying that off-target effects might occur unpredictably. To simultaneously examine the safety and efficiency of these mtDNA cytosine base editor variants, we constructed a 3D coordinate map comparing all three editing types, which revealed that G1333-DdCBE^K1402D/E^ displayed the highest editing efficiency among the 8 candidates with relatively fewer off-target and undesired effects compared to other variants (Fig. 1n). To assess whether the G1333-DdCBE^K1402D/E^ variants showed lower off-target editing when targeting other mitochondrial genome sites, we examined their activity in targeting mND2-site2 (Fig. 1m) and mND4-site2 (Supplementary information, Fig. S5h). The results demonstrated that both G1333-DdCBE^K1402D/E^ variants showed significantly fewer off-target effects in mtDNA than G1333-DdCBE^WT^. While further comparing to the previously reported high-fidelity DdCBE variants (DdCBE^K1389A/T1391A^,^6^ DdCBE^N1308A/Q1310A^ ^2^), we found that G1333-DdCBE^K1402D/E^ appeared to offer the most effective reduction of off-target effects among the variants tested, while presenting comparable or higher editing efficiency than previously reported variants across tested sites (Supplementary information, Fig. S6). In addition, we tested different combinations of double conversion variants at these sites, but found that on-target editing efficiency significantly decreased (Supplementary information, Fig. S7), leading us to focus on the single amino acid variants.

In previous research, we used the high-sensitivity GOTI^7^ method to detect off-target edits, which uncovered surprisingly extensive genome-wide off-target effects in mouse embryos expressing DdCBE^WT^.^1^ We therefore applied GOTI to evaluate the genome-wide off-target effects of the G1333-DdCBE^K1402D/E^ variants that showed high-fidelity targeting in mtDNA (Fig. 1o). Briefly, we injected mRNAs encoding Cre and individual G1333-DdCBE variants into one blastomere of two-cell stage embryos carrying a loxP-Stop-loxP-tdTomato gene, then transplanted the modified embryos into surrogate mice, and isolated tdTomato^+^ and tdTomato^–^ cells from E14.5 embryos by FACS for genomic DNA (gDNA) extraction and whole-genome sequencing (WGS).

Further analysis of WGS data showed that G1333-DdCBE^WT^ introduced approximately 1074 ± 174 off-target single nucleotide variants (SNVs) across the genome (Fig. 1p; Supplementary information, Table. S1), in line with previous reports.^1^ Alignment of all off-target sites from all samples showed no overlap between them, or with off-target sites predicted by TALENoffer, PROGNOS, or BLASTn (Fig. 1q). Moreover, these off-target events were preferentially located in the “TC” context, whereas no such events were found in embryos expressing the G1333-DdCBE^K1402D/E^ variants (Supplementary information, Fig. S8a). Among these variants, off-target SNVs were significantly less frequent compared to G1333-DdCBE^WT^, averaging 5 and 6 in G1333-DdCBE^K1402D/E^, respectively, similar to the Cre-only control group (Fig. 1p). Closer examination of the SNVs identified in the G1333-DdCBE^WT^ group showed a preponderance of G- > A/C- > T nucleotide substitutions, while no clear pattern was detected in embryos expressing G1333-DdCBE^K1402D/E^ (Fig. 1r), further supporting the context independence of the variant off-targets. Analysis of indels in WGS data showed no difference in off-target frequency between any variants and the Cre-only controls (Supplementary information, Fig. S8b). These results highlighted the high fidelity of mtDNA editing by the G1333-DdCBE^K1402D/E^ variants, supporting their further exploration for potential clinical application.

Single-stranded cytosine deaminases, such as BE3, can induce off-target effects in RNAs beyond their activity with gDNA substrates.^8^ Since DddA_tox_ is a double-stranded cytosine deaminase, the rarity of duplex RNAs results in a low likelihood of DdCBEs inducing off-target effects in RNA. To verify that G1333-DdCBEs indeed do not cause off-target effects in RNA or RNA–DNA heteroduplexes with physiological or genetic consequences for the host, we also evaluated off-target events across the whole transcriptome in cells expressing G1333-DdCBE^WT^ or G1333-DdCBE^K1402D/E^. As a result, we detected 595.33 ± 43.8 RNA-SNVs in EGFP vector control group relative to the untransfected control group (Supplementary information, Fig. S9a), while 661.33 ± 15.56 RNA-SNVs were detected in the G1333-DdCBE^WT^ transcriptome, 496 ± 81.55 in G1333-DdCBE^K1402D^ group, 494 ± 62.91 in G1333-DdCBE^K1402E^ group. There were no significant differences among groups. Additionally, the A/T/G/C distribution in SNVs followed a similar pattern among groups (Supplementary information, Fig. S9b, c), indicating a lack of preferential base conversion in RNAs. Collectively, these results suggested that none of the G1333-DdCBE variants introduced substantially more RNA-level SNVs than the vector control.

Although long proposed as a potentially effective therapeutic strategy for mitochondrial diseases, DdCBE development has languished due to high risks of undesired editing and the aforementioned widespread off-target effects. Here, by modifying the DNA-binding capability of DddA_tox_ through charge reversal, we identified the G1333-DdCBE^K1402D/E^ mitochondrial base editors with ultra-low mitochondrial and genome-wide off-target effects, and with a narrower editing window compared to G1333-DdCBE^WT^. It should also be noted that double-stranded base editors used in this study did not induce significant SNV effects in RNA. It might stem from DddA_tox_’s inability to bind to and edit single-stranded RNA or RNA–DNA heteroduplexes. The mtDNA-targeted split architectures described here demonstrate improved safety and reliability for cell- and embryo-level research, providing a foundation to develop treatments for mitochondria-related diseases. In addition, they also indicate a feasible strategy to reduce off-target effects in deaminase base editors.

## Supplementary information

Supplementary Figures and methods

Supplementary Table S1

## Data Availability

All sequencing data were deposited in the NCBI Sequence Read Archive (SRA) under project accession PRJNA1028171.
