# Supplementary material for: Reducing off-target effects of DdCBEs by reversing amino acid charge near DNA interaction sites
Source: Cell Res. 2024 Sep 10;34(12):877–81. doi: 10.1038/s41422-024-01028-w (PMC11615329; doi:10.1038/s41422-024-01028-w)
Supplement: Supplementary file 1 — Supplementary Figures and methods [file 41422_2024_1028_MOESM1_ESM.pdf]

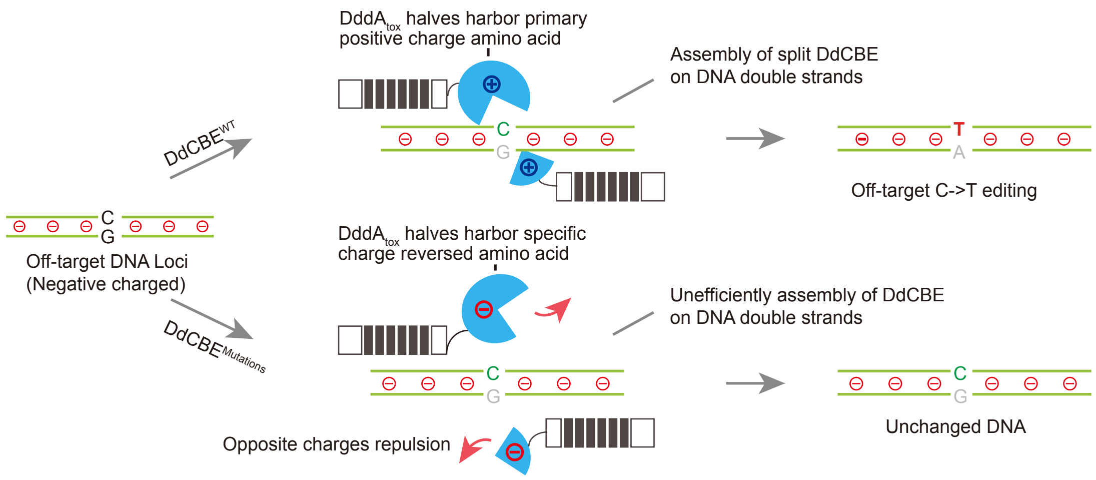

Fig. S1. Schematic for rational engineering of DdCBEs to reduce off-target effects.
In the DdCBEWT, positively charged amino acids on the surface of DddAtox played a crucial role in facilitating its binding to negatively charged double-stranded DNA. Consequently, this interaction resulted in off-target effects at undesired sites. In the improved DdCBE variants, modifications were made by substituting the positively charged amino acids with negatively charged ones. This change weakens the binding capacity of DddAtox to DNA at off-target sites due to reduced affinity, thereby reducing the occurrence of off-target events.

a

G1333-DddAtoxin-N/C

1290 GSYALGPYQISAPQLPAYNGQTVGTFYYVNDAGGLESKVFSSGGPTPYPNYANAGHVEGQS
1351 ALFMRDNGISEGLVFHNNPEGTCGFCVNMTETLLPENAKMTVVPPEGAIPVKRGATGETKVF
1413 TGNSNSPKSPTKGGC

G1397-DddAtoxin-N/C

1290 GSYALGPYQISAPQLPAYNGQTVGTFYYVNDAGGLESKVFSSGGPTPYPNYANAGHVEGQS
1351 ALFMRDNGISEGLVFHNNPEGTCGFCVNMTETLLPENAKMTVVPPEGAIPVKRGATGETKVF
1413 TGNSNSPKSPTKGGC

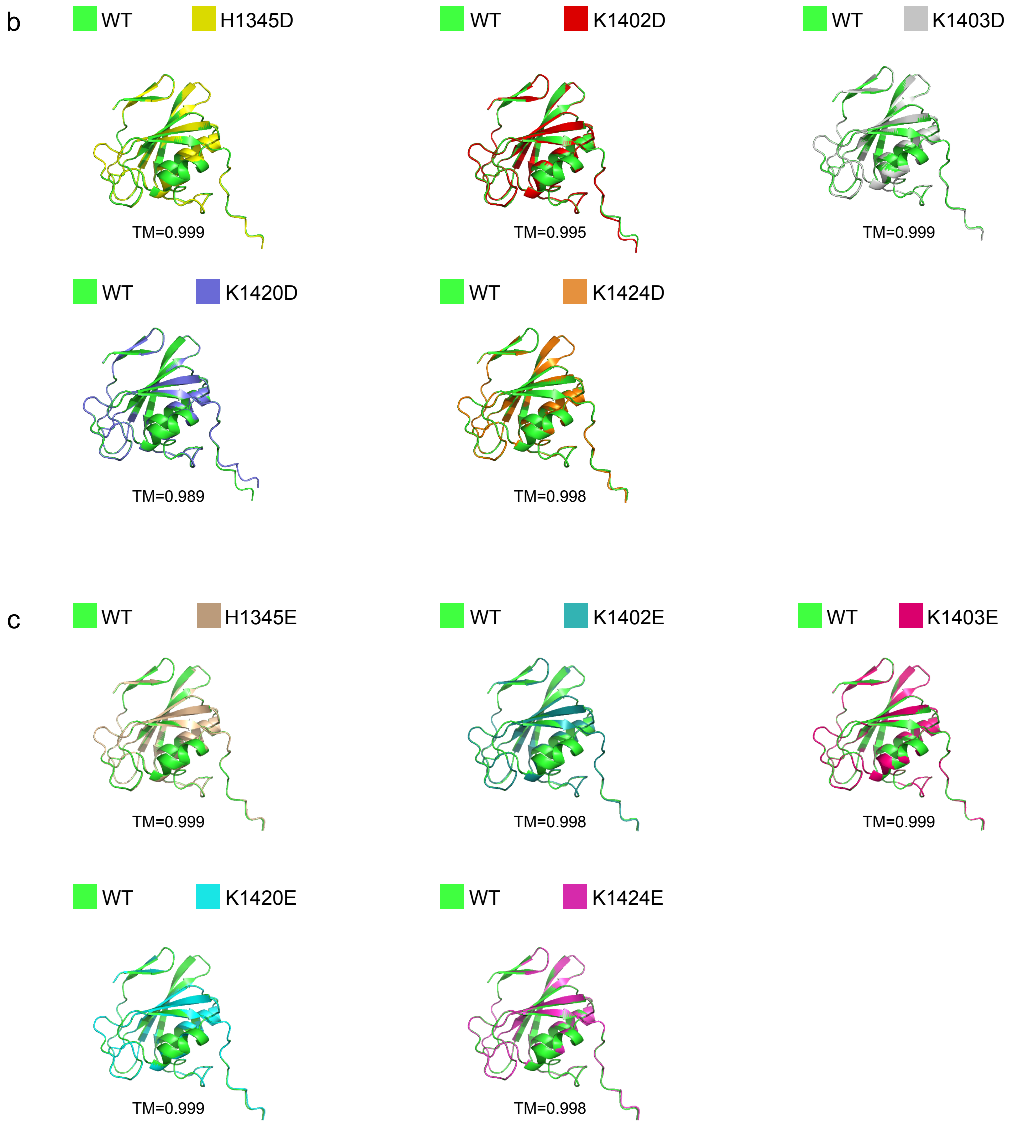

Fig. S2. Single amino acid mutations on the DNA interacting surface do not affect the 3D structure of DddAtox variants.

a, Positively charged amino acids on the DddAtox surface potentially interacting with DNA in G1397- and G1333-split forms. **b-c**, Substitution of positively charged amino acids on the DddAtox surface with aspartic acid (**b**) or glutamic acid (**c**) does not significantly alter the structure of the DddAtox protein.

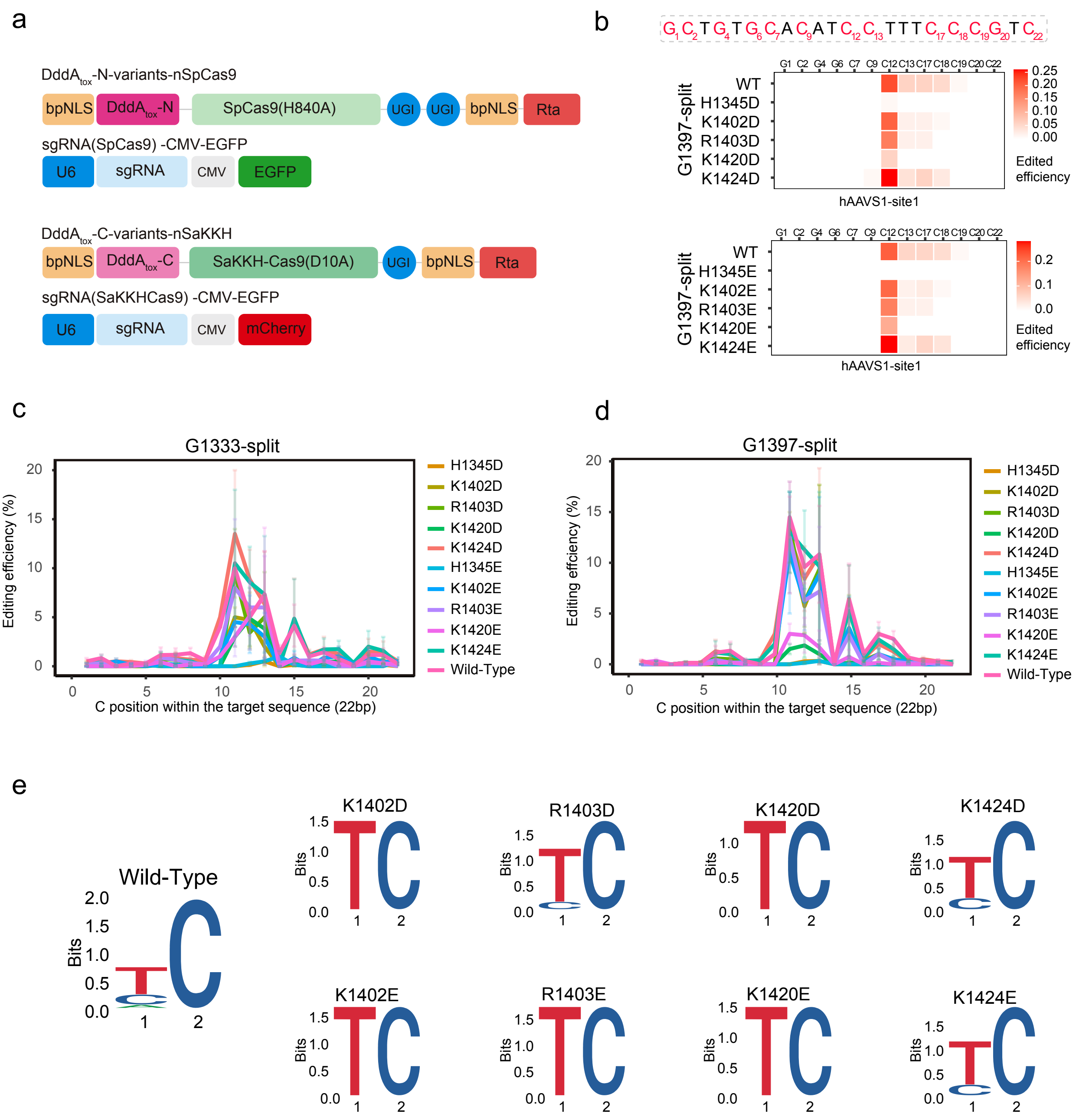

Fig. S3. Cytosine base editing characteristics of the DddAtox variants in a split-DddAtox-Cas9 fusion architecture.

a, Plasmid architectures of split-DddAtox–Cas9 fusions. DddAtox-N and DddAtox-C terminals were fused to nSpCas9(H840) and nSaKKHCas9(D10A), respectively. **b**, Heat maps of C/G-to-T/A conversion frequency for D/E variants split at the G1333 or G1397 hAAVS1 sites. **c-d**, The frequencies of C/G-to-T/A conversion at different C bases within the target sequence of G1333- (**c**) and G1397-split-DddAtox-Cas9 (**d**) fusion variants. **e-f**, Preferential sequence context for cytidine editing in the Wild-Type (**e**) and mutated (**f**) G1333/G1397-split-DddAtox-Cas9 .

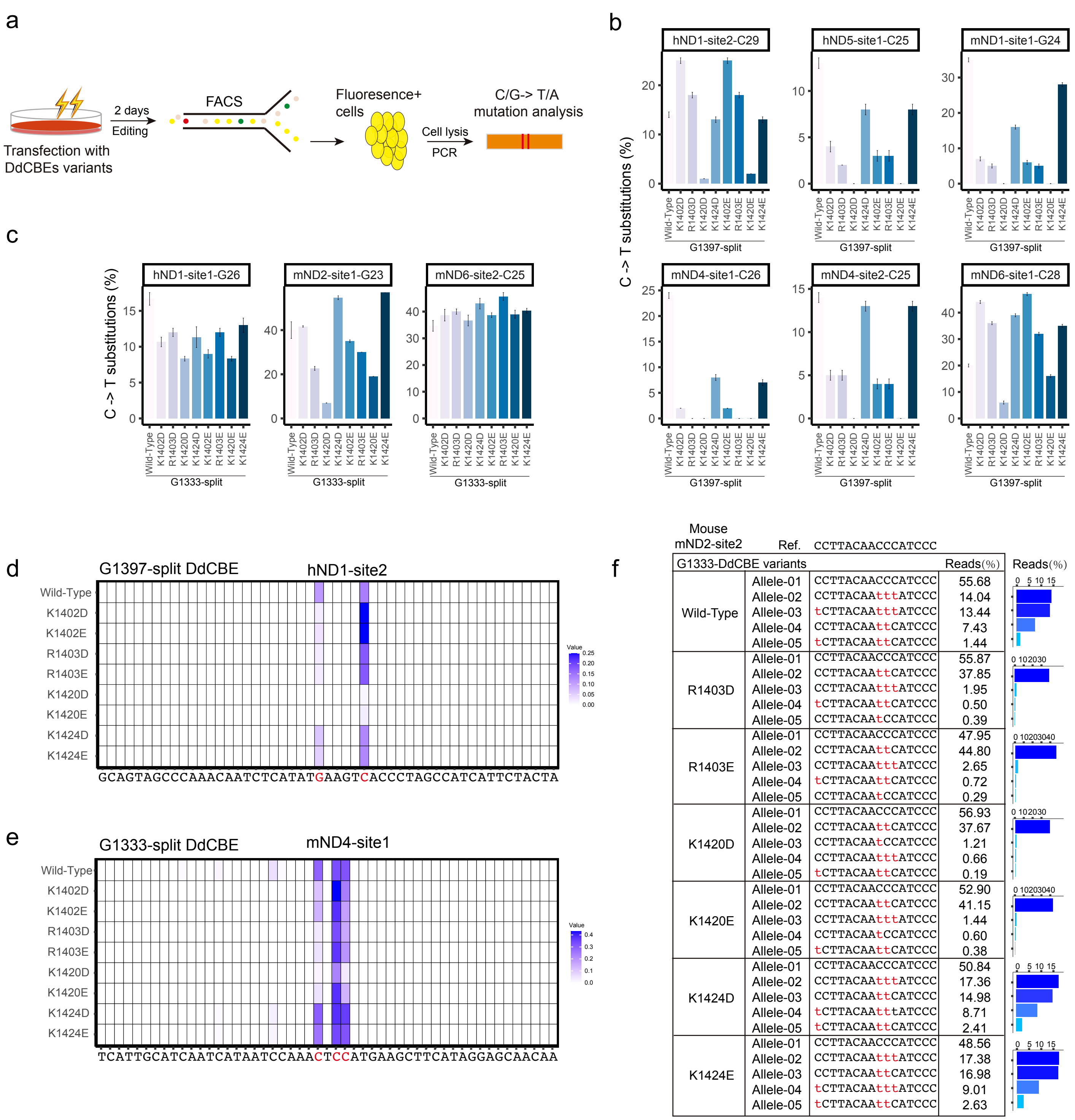

Fig. S4. Cytosine base editing by DddAtox variants in the human and mouse mitochondrial genome. **a**, Experimental scheme for detecting DdCBE efficiency. **b**, Editing efficiency of G1397-DdCBE variants at human and mouse mitochondrial genome sites. **c**, Editing efficiency of G1333-DdCBE variants in human and mouse mitochondrial genome sites. **d**, Detailed characteristics of G1397-DdCBE variant base editing activity at hND1-site2. **e**, Detailed characteristics of G1333-DdCBE activity at mND4-site1 and mND2-site2. **f**, Edited alleles of G1333-DdCBE variants at mND2-site2. All *P* values were determined by two-sided Student's *t*-tests. $n \geq 3$ replicates were used in all experiments.

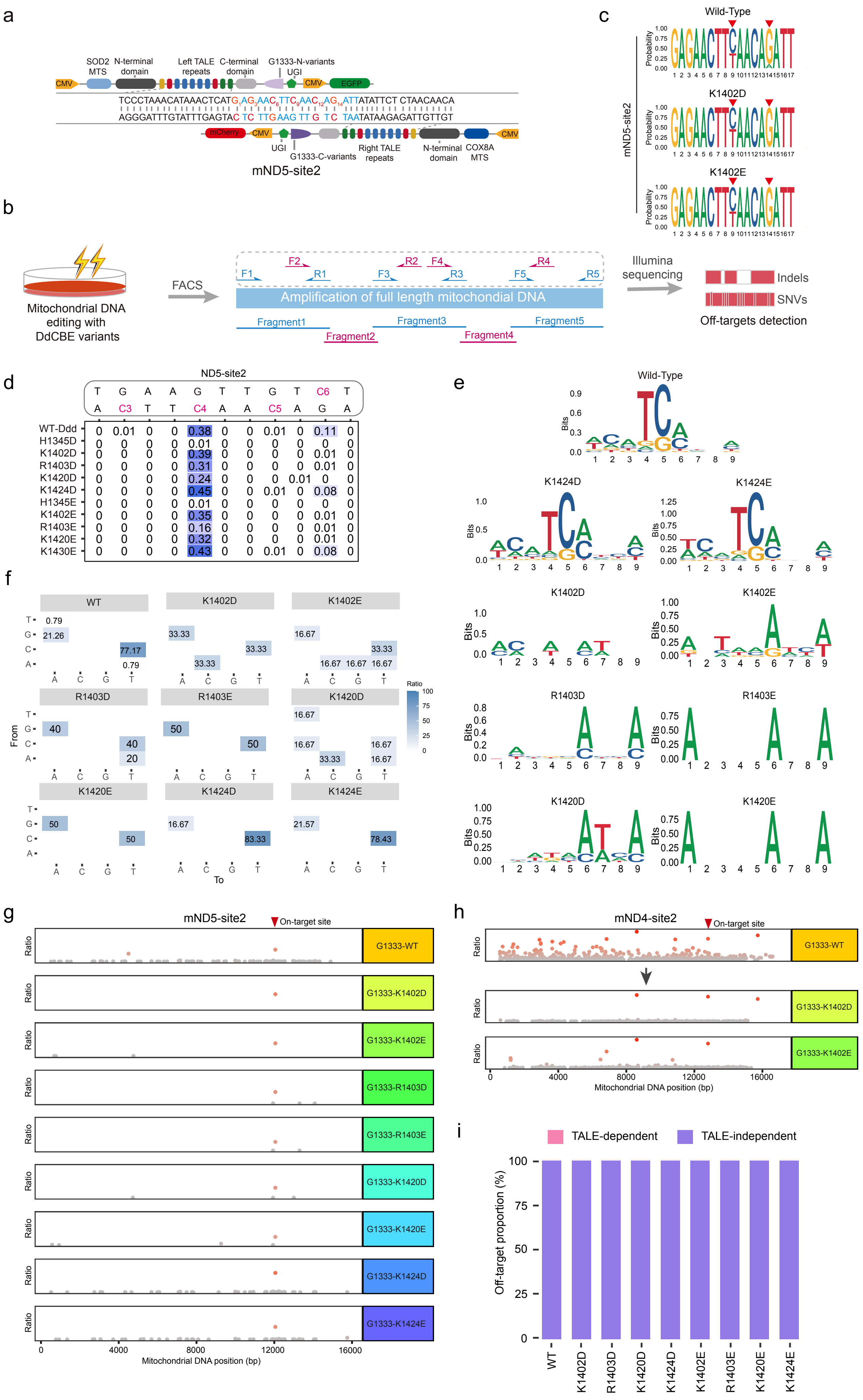
a
SOD2 MTS
N-terminal domain
Left TALE repeats
C-terminal domain
G1333-N-variants
UGI
CMV
EGFP
TCCCTAAACATAAACTCATGAGAACTTCAACAGATTATATTCT CTAACAACA
AGGGATTTGTATTTGAGTAC TC TTGAAG TT G TC TAATATAAGAGATTGTTGT
mCherry
G1333-C-variants
Right TALE repeats
N-terminal domain
COX8A MTS
mND5-site2
b
Mitochondrial DNA editing with DdCBE variants
FACS
F1 F2 R1 F3 R2 F4 R3 F5 R4 R5
Amplification of full length mitochondrial DNA
Fragment1 Fragment2 Fragment3 Fragment4 Fragment5
Illumina sequencing
Indels
SNVs
Off-targets detection
c
Wild-Type
K1402D
K1402E
mND5-site2
Probability
d
ND5-site2
T G A A G T T G T C6 T
A C3 T T C4 A A C5 A G A
WT-Ddd 0 0.01 0 0 0.38 0 0 0.01 0 0.11 0
H1345D 0 0 0 0 0.01 0 0 0 0 0 0
K1402D 0 0 0 0 0.39 0 0 0 0 0.01 0
R1403D 0 0 0 0 0.31 0 0 0 0 0.01 0
K1420D 0 0 0 0 0.24 0 0 0 0.01 0 0
K1424D 0 0 0 0 0.45 0 0 0.01 0 0.08 0
H1345E 0 0 0 0 0.01 0 0 0 0 0 0
K1402E 0 0 0 0 0.35 0 0 0 0 0.01 0
R1403E 0 0 0 0 0.16 0 0 0 0 0.01 0
K1420E 0 0 0 0 0.32 0 0 0 0 0.01 0
K1430E 0 0 0 0 0.43 0 0 0.01 0 0.08 0
e
Wild-Type
K1424D
K1424E
K1402D
K1402E
R1403D
R1403E
K1420D
K1420E
Bits
f
WT
K1402D
K1402E
R1403D
R1403E
K1420D
K1420E
K1424D
K1424E
From
To
Ratio
g
mND5-site2
On-target site
Ratio
G1333-WT
G1333-K1402D
G1333-K1402E
G1333-R1403D
G1333-R1403E
G1333-K1420D
G1333-K1420E
G1333-K1424D
G1333-K1424E
Mitochondrial DNA position (bp)
h
mND4-site2
On-target site
G1333-WT
G1333-K1402D
G1333-K1402E
Mitochondrial DNA position (bp)
i
TALE-dependent
TALE-independent
Off-target proportion (%)
WT
K1402D
R1403D
K1420D
K1424D
K1402E
R1403E
K1420E
K1424E

Fig. S5. Assessment of off-target effects in the mitochondrial genome for DddAtox variants with altered surface charge.

a, Schematic for detecting off-target effects of DdCBEs across the whole mitochondrial genome.
b, Architecture of G1333-DdCBE variants targeting the mND5-site2. **c**, The editing context of undesired C/G-to-T/A conversion of DdCBE^{WT}, DdCBE^{K1424D}, and DdCBE^{K1424E} variants. **d-e**, The "TC" feature were found in the context around off-target C/G loci of DdCBE^{WT} and DdCBE$^{K1424D/E}$ (**d**), but not in DdCBE$^{K1402D/E}$, DdCBE$^{R1403D/E}$, or DdCBE$^{K1420D/E}$ (**e**). **f**, off-target SNV nucleotide substitution patterns of different DdCBE variants in mitochondrial base editing. **g**, The off-target loci of G1333-DdCBE variants in mND5-site2.
h, The off-target loci of G1333-DdCBE^{WT}, G1333-DdCBE$^{K1402D/E}$ variants in mND4-site2. **i**, The majority of off-target sites for different DdCBE variants are TALE-independent.

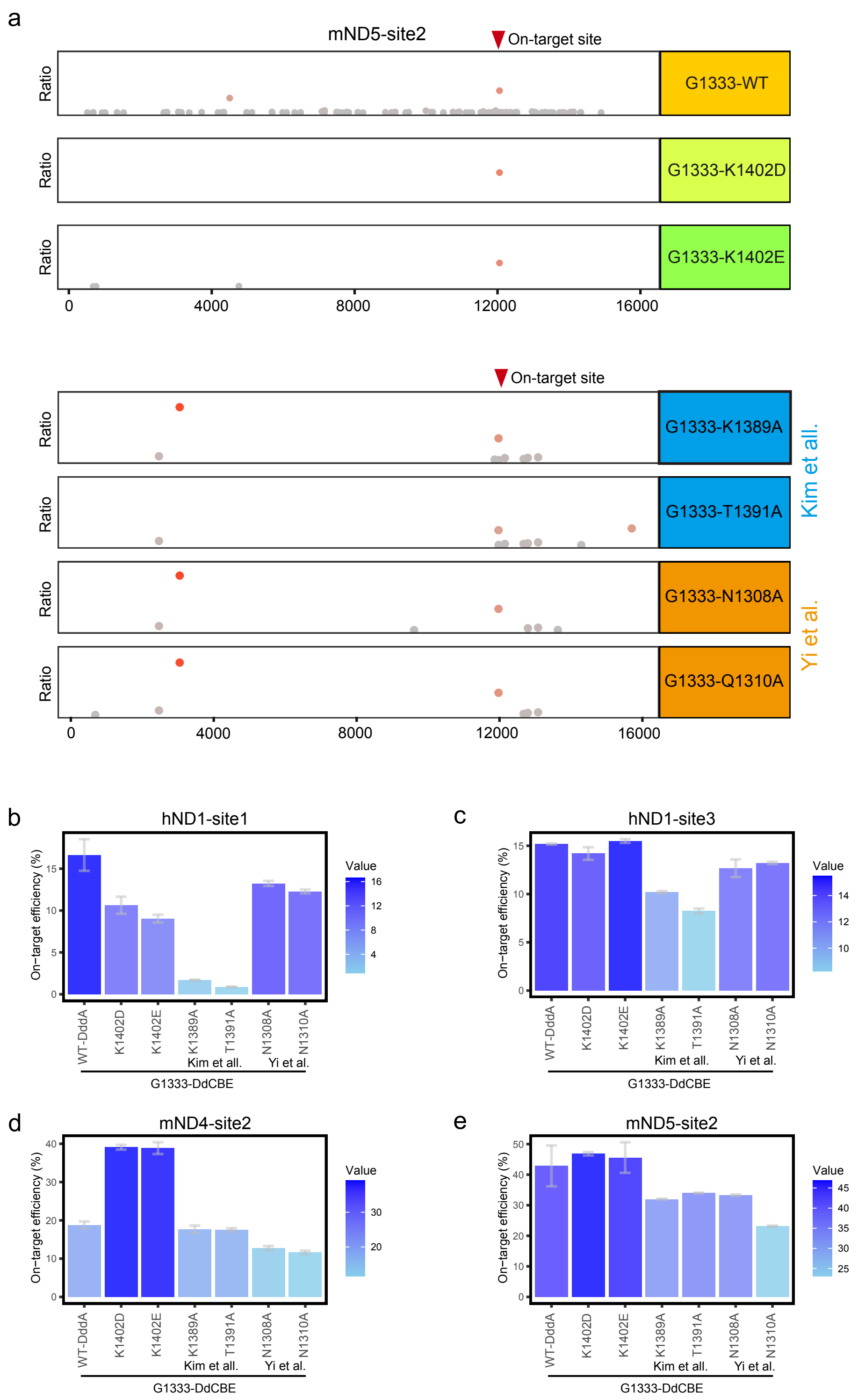

Fig. S6. Editing effects and off-target analysis of G1333-DdCBE variants in mitochondrial genomes. **a**, Distribution of off-target loci in the mitochondrial genome induced by different DdCBE variants. The K1389A/T1391A DdCBE variants were reported by Kim et al. [6], while the N1308A/Q1310A variants were reported by Yi et al [2]. **b-e**, On-target efficiency of different DdCBE variants using the G1333-split form at hND1-site1 (**b**), hND1-site3 (**c**), mND4-site2 (**d**), and mND5-site2 (**e**) loci.

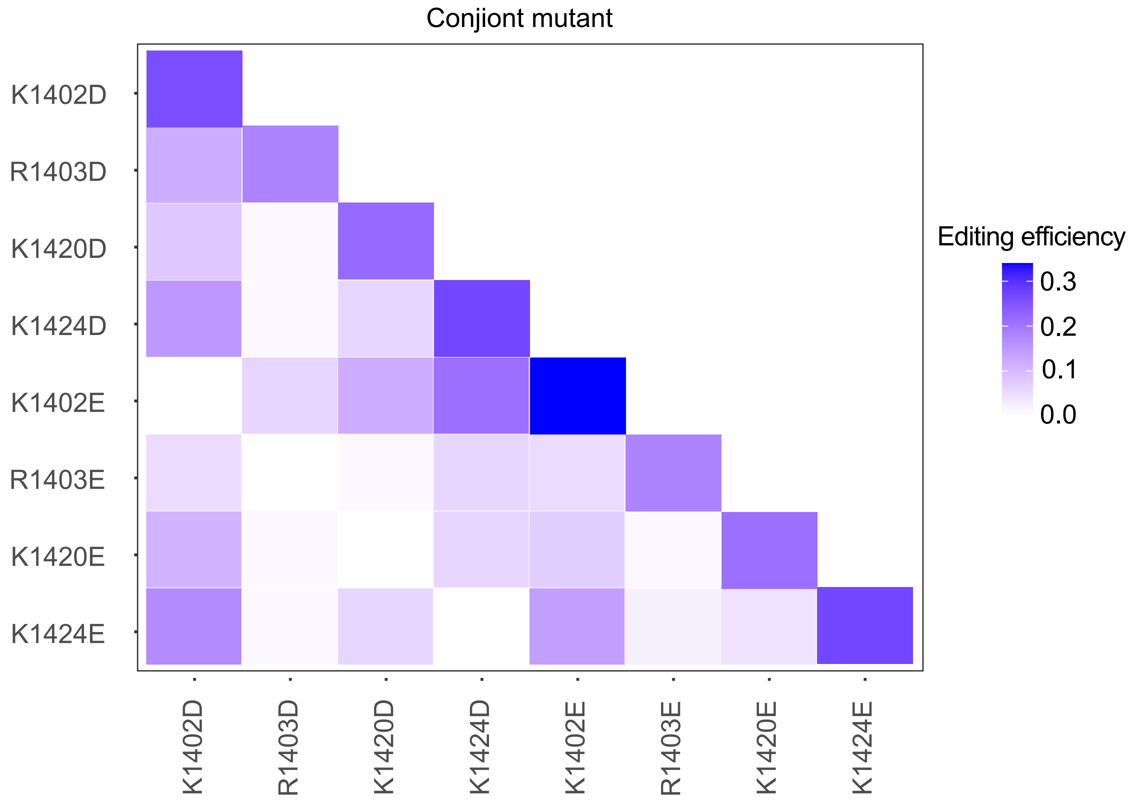

Fig. S7. Decreased on-target efficiency of conjiont mutant DdCBEs.

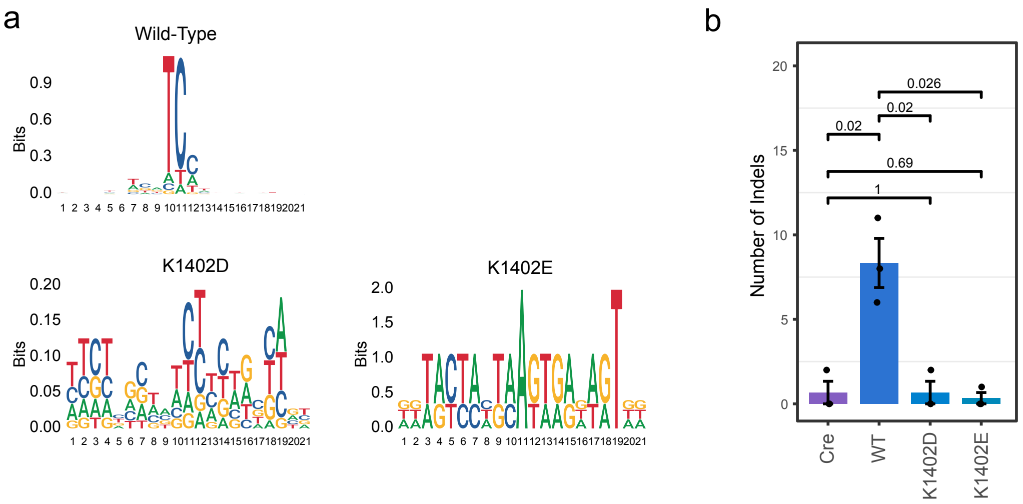

Fig. S8. Assessment of genome-wide off-target effects of DdCBEK1402D/E and DdCBEK1420E. **a**, Seuquence context around SNV mutation site for DdCBEWT and DdCBEK1402D/E. **b**, Quantification of off-target edits resulting in insertions/deletions (Indels) for WT and split DdCBEs variants. All *P* values were calculated by two-sided Student's *t*-tests.

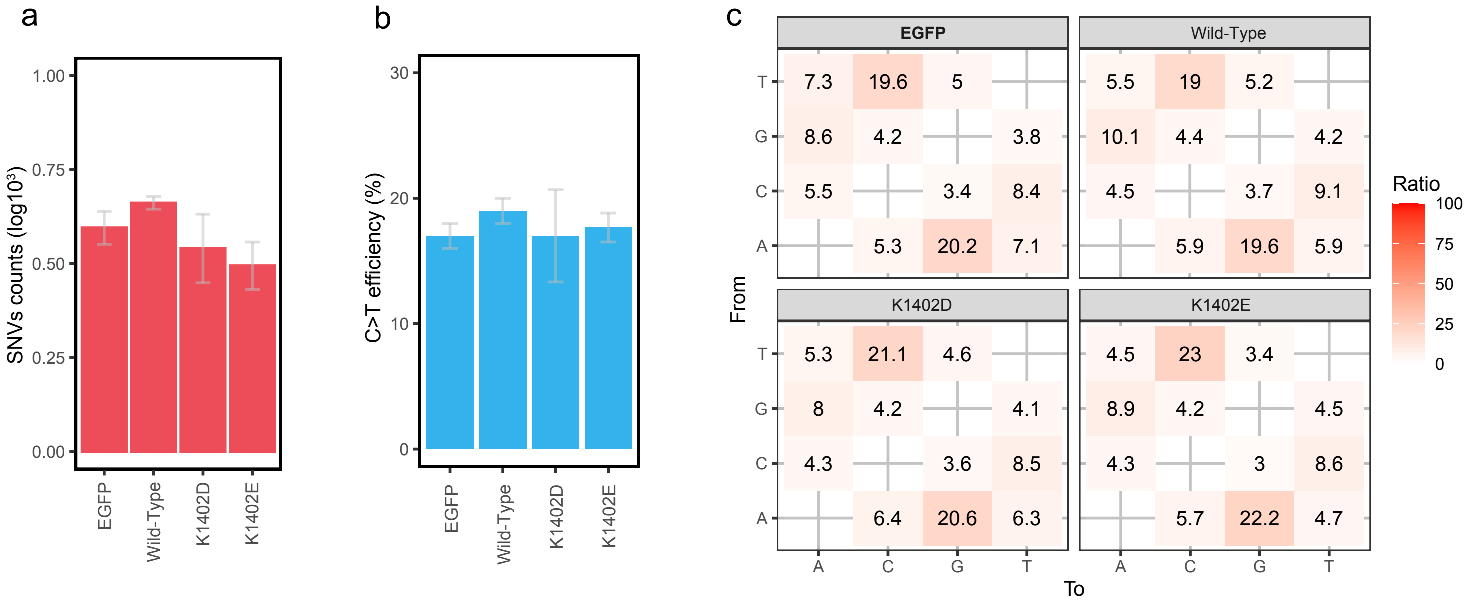

Fig. S9. Assessment of RNA-level off-target effects for split variant DdCBEs.
a, Abundance of SNVs off-target edits (SNVs) in RNA in N2a cells treated with the different DdCBE variants. **b**, The nucleotide frequency of RNA-SNVs from different DdCBE variants groups. **c**, Patterns of base conversion in each DdCBE variants group. In panels b and c, the nucleotide frequency and conversion pattern analysis were performed on the total SNVs derived from repeats ($n \geq 3$). All P values were calculated by two-sided Student's t-tests. $n \geq 3$ replicates were used in all experiments.

Material and Method

Animal care

Embryos were collected from 4-week-old female C57BL/6 mice and homozygous Ai9 (B6.Cg-Gt(ROSA)26Sortm9(CAG-tdTomato) Hze/J; JAX strain 007909) male mice. ICR females were chosen to serve as recipients. All animals involved in the study were cared for in accordance with the guidelines established by the Biomedical Research Ethics Committee of Agricultural Genomics Institute in Shenzhen, Chinese Academy of Agricultural Sciences.

Plasmid construction

In the case of TALE plasmid, the TALE module were assembely using the method of Golden Gate[9], and the $DddA_{tox}$-N/C variants were modified using PCR amplification using primers that contained mutation sites. Subsequently, they were fused to the C-terminal of TALE. Additionally, an UGI component was introduced to the C-terminal of $DddA_{tox}$-N/C. For DddA-N-SpCas9 and DddA-C-SaCas9 plasmids, the plasmid backbone was generously gifted by Yunbo Qiao from Shanghai Jiao Tong University. The connection between the $DddA_{tox}$-N/C variants and the N-terminal of the backbone was achieved through the utilization of a 32aa linker and the NEBuilder HiFi DNA Assembly Cloning Kit (NEB), following the instructions provided. For sgRNA plasmids construction, the sgRNA oligos were annealed and inserted before the scaffold through T4 ligations. All these plasmids were extracted according to the instructions provided in the E.Z.N.A Endo-free plasmid mini kit manuals.

Cell culture, transfection and genotyping

HEK293T cells were cultured and maintained at 37 °C in 5% CO_2. Cells were grown in high glucose DMEM supplemented with 10% (v/v) fetal bovine serum (BI). Mouse N2a cells were cultured in high glucose DMEM supplemented with 10% (v/v) fetal bovine serum (Gibco) and maintained at 37 °C in 5% CO_2. For transfection, cells were plated in 12 wells culture plates at a density of 2×10^5 cells per well, 18-24h before transfection. 1.2 ug of $DddA_{tox}$-N-SpnCas9 plasmid, 1.2 ug of $DddA_{tox}$-C-SanCas9 plasmid, 0.8ug of each sgRNA were used to make up 4ug of total plasmid DNA and co-transfected into HEK293T cells via polyethylenimine (PEI). In N2a cells, 2 ug of each TALE half monomer plasmid were co-transfected using Lipo8000 transfection reagent (Beyotime Biotechnology). Cells were collected on day four post transfection for genotyping.

To perform the on-target assay, we sorted 10,000 EGFP+/mCherry+ cells using FACS and transferred them into lysis buffer (0.1% Triton X-100, 0.1% TWEEN20, 4ug/mL proteinase K). Then, we lysed the cells using a PCR program with the following steps: incubation at 55°C for 30 minutes, heating to 95°C for 10 minutes, and holding at 4°C. Nest PCR was performed to amplified the target region sequence from gDNA for Sanger sequencing or further high-throughput sequencing.

Mitochondrial DNA off-target detection

To analyze the potential off-target effects of DdCBEs on mtDNA, we employed the following experimental steps. Firstly, we isolated 50,000 cells using FACS and lysed them with a lysis buffer. The entire length of the mtDNA was then amplified using nested PCR, which involved the utilization of five sets of primers. In the initial round of nested PCR, we amplified the entire mtDNA using five pairs of primers within the same tubes. Subsequently, 10 ul of the PCR products were

used for the second round of PCR amplification in five separate reaction mixes. Each reaction mix used specific PCR primers targeting different regions of the mtDNA.

Following the amplification step, the PCR products was mixed together and fragmented to an average length of 150 bp using a Covaris S200 instrument. The fragmented mtDNA was then subjected to end-repair, A-tailing, and ligation to adapters. Subsequently, purification was performed using AMPure XP (Beckman Coulter) in accordance with the manufacturer's instructions. The prepared mtDNA samples were subsequently subjected to Illumina sequencing.

The obtained NGS sequencing data was aligned to the mitochondrial genome using BWA (0.7.17), while SNV calling was conducted using Mutect2 (v4.2.6). To strictly control the quality of the variants, we removed SNV reported in dbSNP150 database. Finally, R (v4.1.2) was utilized for generating figures based on the obtained results.

GOTI experimental design

The GOTI assays were conducted in a similar manner to our previous report[7]. In summary, a mixture of mRNA was injected into one blastomere of a two-cell stage embryo that was produced by crossing wild-type female mice with Ai9 male mice. The embryos were then transplanted into surrogate C57 mice 2-4 hours post-injection. At E14.5, tdTomato+ and tdTomato- cells were collected from the transplanted embryos using FACS. Three variant calling algorithms (Mutect2, Scalpel, and Strelka for Indels; Mutect2, Lofreq, and Strelka for SNVs) were used to identify off-target Indels and SNVs by comparing the two types of cells. Sequencing coverage at off-target sites was subsequently confirmed using Integrative Genomics Viewer (IGV).

Generation of mRNA

For the in vitro transcription of mRNA, TALE plasmids including a T7 promoter and corresponding architectures (different $DddA_{tox}$ variants) were respectively linearized as temples. For Cre, the T7 promoter was added to the N-terminal of Cre coding region by PCR amplification using the following primer:

Name	Sequence (5'-3')
Cre IVT F	TAATACGACTCACTATAGGGAGACAGATCACCTTTCCTATCAACC
Cre IVT R	TCGGTATTTCCAGCACACTGGA

PCR products were purified and used as temples for IVT. MESSAGE mMACHINE T7 ULTRA kit (Thermofisher) was used for the above architecture mRNA generation. All IVT products of mRNA were purified using the MEGA clear kit (Thermofisher) and eluted in RNase-free water.

2-cell embryo injection, embryo culturing, and embryo transplantation

To collect embryos, C57BL/6 females underwent preliminary superovulation and were mated with homozygous Ai9 males 24 hours after hCG injection. Fertilized embryos were then obtained from the oviducts. For 2-cell embryo injection, all TALE groups were prepared at a concentration of 100ng/μL and co-injected with Cre mRNA (2ng/μL) into one blastomere of 2-cell embryos 48 hours post hCG injection. During the injection process, embryos were placed in a droplet of M2 medium

supplemented with 5ug/mL cytochalasin B (CB), and a constant flow setting was applied using the FemtoJet microinjector (Eppendorf). The injected embryos were subsequently cultured in KSOM medium with amino acids at 37 °C under 5% CO_2 for 2 hours before being transferred into the oviducts of pseudo-pregnant ICR females at 0.5 dpc.

Embryo genotyping

For embryo genotyping, single blastocyst at E4.5 was transferred into 4ul of lysis buffer containing 0.1% Triton X-100, 0.1% TWEEN20, and 4ug/mL proteinase K. The following program was executed to expose DNA: incubation at 55 °C for 30 minutes, followed by heating to 95 °C for 10 minutes, and then cooling to 4 °C indefinitely. To perform embryo cell genotyping, nested PCR targeting loci was conducted. In the first round of PCR amplification, Extaq (Takara) was activated at 95 °C for 3 minutes. Subsequently, PCR was carried out for 30 cycles with denaturation at 95 °C for 30 seconds, annealing at 55 °C for 30 seconds, extension at 72 °C for 1 minute, and a final extension step at 72 °C for 5 minutes after the cycles. For the second round of PCR, the same program was applied using inner nested primers. The PCR products were then purified for Sanger Sequencing to determine the genotyping results.

FACS

At E14.5, embryos were used to obtain tissues for further analysis. The embryo tissues were dissociated into small pieces and then digested in 5 mL of Trypsin-EDTA solution (0.05%) at a temperature of 37°C for 30 minutes. To stop the digestion process, 5 mL of DMEM medium supplemented with 10% Fetal Bovine Serum (FBS) was added. The fetal tissues were homogenized by pipetting 30-40 times using a 1 mL pipette tip. Afterwards, the homogenized tissue suspension was centrifuged at 1000g for 6 minutes. The supernatant was discarded, and the pellet containing the tissue cells was re-suspended in DMEM medium supplemented with 10% FBS. Following the suspension step, the cell suspension underwent filtration through a 40μm cell strainer to obtain a single-cell suspension. The tdTomato-positive (tdTomato+) and tdTomato-negative (tdTomato-) cells were then isolated using Fluorescence-Activated Cell Sorting (FACS).

Whole genome sequencing (WGS) and data analysis

In this study, whole genome sequencing (WGS) was performed on the DNA extracted from tdTomato+/tdTomato- cells. The DNeasy Blood and Tissue Kit (catalog number 69504, Qiagen) was used for DNA extraction following the manufacturer's instructions. For WGS, BGI DNBSEQ-T7 platform was utilized, with an average coverage of 50x. The sequencing reads obtained were then mapped to the reference genome (GRCm39) using BWA (v0.7.17). Picard tools (v2.25.7) were employed to sort and mark duplicates in the resulting mapped BAM files. To identify genetic variations, Strelka (v2.9.10) was initially utilized for the detection of de novo insertions and deletions (Indels) as well as single nucleotide variants (SNVs) across the entire genome. Candidate regions were selected by considering 200 base pairs upstream and downstream of the mutation locations. Mutect2 (v4.2) and Lofreq (v2.1.5) algorithms were then employed to detect SNVs within the candidate regions, while Mutect2 and Scalpel (v0.5.4) were used to detect Indels within the same regions. Variants detected by all three algorithms were considered true SNVs or Indels. The tdTomato- data served as a control to identify mutations specific to the tdTomato+ data in each pair of samples. Additionally, manual realignment and removal of variant loci within repeat sequence

arrays were performed to confirm and refine the identified SNVs and Indels. In addition, to predict the off-target sites, TALENoffer[2] (http://www.jstacs.de/index.php/TALENoffer) was applied in different TALE group, the top 100 off-target sites overlapped with the Indels or SNVs detected in GOTI examinations.

RNA sequencing and data analysis

To analyze the potential RNA off-target effects of DdCBEs, we followed these experimental steps. First, we isolated 200,000 mCherry+/EGFP+ N2a cells using FACS and proceeded with RNA extraction. Next, high-throughput mRNA sequencing was performed using the BGISeq500 platform. SOAPnuke (v2.1.7) was used for quality control. Qualified reads were mapped to the mouse reference genome(mm39) by STAR (v2.7.10b) with two pass model. The variants were called by GATK (v4.2.6.1). To identify variants with high confidence, we filtered clusters of at least 5 SNVs that were within a window of 35 bases and retained variants with base-quality score >25, mapping quality score >20, Fisher strand values >30.0, qual by depth values <2.0 and sequencing depth >20. In addition, we filtered variants detected in the WT sample, and those in the dbSNP150 dataset. Finally, R (v4.1.2) was utilized for generating figures based on the obtained results.

Statistical analysis

Statistical analyses in this study were conducted using R version 4.2.1 (https://www.r-project.org). All tests were performed as two-sided, and a significance level of $P < 0.05$ was used to determine statistical significance.
